# LINC00160 mediated paclitaxel‐And doxorubicin‐resistance in breast cancer cells by regulating TFF3 *via* transcription factor C/EBPβ

**DOI:** 10.1111/jcmm.15487

**Published:** 2020-07-11

**Authors:** Huaiguo Wu, Juan Gu, Daoping Zhou, Wei Cheng, Yueping Wang, Qingping Wang, Xuedong Wang

**Affiliations:** ^1^ Center for Precision Medicine Anhui No.2 Provincial People’s Hospital Hefei China; ^2^ Department of Medical Laboratory Science The Fifth People’s Hospital of Wuxi Nanjing Medical University Wuxi China; ^3^ Department of Pathology The Fifth People’s Hospital of Wuxi The Medical School of Jiangnan University Wuxi China; ^4^ Department of Biology College of Arts & Science Massachusetts University Boston MA USA

**Keywords:** breast cancer, C/EBPβ, drug resistance, long non‐coding RNA‐00160, TFF3

## Abstract

Chemoresistance represents a major challenge in breast cancer (BC) treatment. This study aimed to probe the roles of LINC00160 in paclitaxel‐ and doxorubicin‐resistant BC cells. Three pairs of BC and adjacent normal tissue were used for lncRNA microarray analysis. Paclitaxel‐resistant MCF‐7 (MCF‐7/Tax) and doxorubicin‐resistant BT474 (BT474/Dox) cells were generated by exposure of parental drug‐sensitive MCF‐7 or BT474 cells to gradient concentrations of drugs. Correlation between LINC00160 expression and clinical response to paclitaxel in BC patients was examined. Short interfering RNAs specifically targeting LINC00160 or TFF3 were designed to construct LINC00160‐ and TFF3‐depleted BC cells to discuss their effects on biological episodes of MCF‐7/Tax and BT474/Dox cells. Interactions among LINC00160, transcription factor C/EBPβ and TFF3 were identified. MCF‐7/Tax and BT474/Dox cells stable silencing of LINC00160 were transplanted into nude mice. Consequently, up‐regulated LINC00160 led to poor clinical response to paclitaxel in BC patients. LINC00160 knockdown reduced drug resistance in MCF‐7/Tax and BT474/Dox cells and reduced cell migration and invasion. LINC00160 recruited C/EBPβ into the promoter region of TFF3 and increased TFF3 expression. LINC00160‐depleted MCF‐7/Tax and BT474/Dox cells showed decreased tumour growth rates in nude mice. Overall, we identified a novel mechanism of LINC00160‐mediated chemoresistance via the C/EBPβ/TFF3 axis, highlighting the potential of LINC00160 for treating BC with chemoresistance.

## 1 INTRODUCTION

Breast cancer (BC) represents the most frequently occurring carcinoma among females globally.^1^ The treatment regimens include surgical resection, chemotherapy, radiation therapy and integrative therapies.^2^, ^3^ Resistance to chemotherapy continues to hinder the treatment of patients suffering from BC.^4^ Paclitaxel and doxorubicin are widely used first‐line chemotherapeutic agents, which substantially reduce the risk of recurrence and mortality in BC.^5^, ^6^ Unfortunately, the chemoresistance to anticancer drugs has been widely observed in BC management. When the cells are exposed to accumulating doses of cytotoxic doxorubicin, the multidrug‐resistant phenotype of cells will develop, and the doxorubicin‐resistant cells commonly manifest notable cross‐resistance to other chemotherapeutic drugs.^7^ The resistance to the chemotherapeutic agent paclitaxel also contributes to unfavourable treatment outcomes.^8^ Recently, long non‐coding RNAs (lncRNAs) have emerged as gene expression regulators of phenotypes of cancer cells and represent promising therapeutic targets in the treatment of chemoresistance in BC.^9^

LncRNAs are abnormally expressed in various human diseases, playing critical roles in promoting pathogenesis or maintaining progression.^10^ Recent evidence has revealed that lncRNAs have the potential to serve as promising biomarkers for the resistance of cancer cells to chemotherapeutic agents, including BC cells.^11^, ^12^ LINC00160 has been shown as a prognostic factor linking to poor BC survival.^13^ LncRNAs‐guided recruitment of transcription factors (TFs) has been recently studied in the chemotherapeutic drug resistance of BC cells. In the LncMAP database (http://bio‐bigdata.hrbmu.edu.cn/LncMAP/), CCAAT‐enhancer binding protein β (C/EBPβ) is a putative TF regulated by LINC00160, and trefoil factor 3 (TFF3) is the downstream target gene of C/EBPβ. C/EBPβ, mostly observed in oestrogen receptor‐negative and highly proliferative and metastatic mammary tumours, appears to be a contributor to tumorigenesis in breast and is usually associated with a poor prognosis.^14^ Additionally, the mRNA expression of TFF3 has been observed to be increased in the blood from metastatic BC patients as compared to that from non‐metastatic patients.^15^ Abnormally expressed TFF3 is involved in the progression of cancers, which accelerates the oncogenic characteristics of prostate cancer cells and diminishes sensitivity to radiation.^16^ Therefore, we hypothesized that the LINC00160/C/EBPβ/TFF3 axis has a regulatory role in the chemoresistance of BC cells.

## 2 MATERIALS AND METHODS

### 2.1 Ethical statement

This study was approved by the ethics committee of Anhui No.2 Provincial People's Hospital. All subjects signed the informed consents. Animal experiment strictly conformed to the relevant regulations of animal experiment management and got the approval of the ethics committee of animal experiment of Anhui No.2 Provincial People's Hospital.

### 2.2 Tissue specimens

BC biopsy specimens and adjacent normal tissue were obtained from 47 female patients (aged 45.60 ± 3.52) (See Table S1 for other basic clinical characteristics) admitted into the Anhui No.2 Provincial People's Hospital between January 2012 and January 2013, and the end time of follow‐up was from January 2017 to January 2018. Inclusion criteria: (1) no radiotherapy or chemotherapy before admission; (2) appropriate for surgical treatment; and (3) no systemic inflammatory response. Exclusion criteria: (1) patients with recurrent BC; (2) with other tumours; and (3) received anti‐inflammatory treatment and anti‐infection treatment 5 months before admission. Each patient was given 175 mg/m^2^ paclitaxel once every three weeks. The follow‐up lasted for a period of 60 months. The overall survival (OS) of patients was defined as the time from the initial date of treatment to date of death. Three pairs of BC and adjacent normal tissue were used for lncRNA microarray analysis. Total RNA from each sample was quantified using the NanoDrop ND‐1000 (Thermo Scientific, Wilmington, USA).

### 2.3 BC cell lines

Human breast epithelial cells MCF10A and human BC cell lines MCF‐7 and BT474 were obtained from American Type Culture Collection (ATCC, Manassas, VA, USA). Paclitaxel‐resistant MCF‐7 (MCF‐7/Tax) cells and doxorubicin‐resistant BT474 (BT474/Dox) cells were generated by continuous exposure of the parental drug‐sensitive MCF‐7 or BT474 cells to gradient concentrations of drugs (Sigma‐Aldrich, St Louis, MO, USA).^17^ Cells were maintained in Dulbecco's modified Eagle's medium supplemented with 10% foetal bovine serum, 100 units/mL penicillin and 100 mg/mL streptomycin (Invitrogen, Carlsbad, CA, USA) in an atmosphere of 5% CO_2_ at 37℃.

### 2.4 RNA interference and overexpression

Short interfering RNAs (siRNAs) specifically targeting LINC00160 and TFF3 were designed to construct LINC00160‐ and TFF3‐depleted BC cells, respectively, with scramble siRNA served as negative controls. Expression vectors containing LINC00160 or TFF3 were introduced for construction of LINC00160‐ or TFF3‐overexpressed BC cells. All vectors were purchased from Shanghai GenePharma Co., Ltd (Shanghai, China). Transient transfections were performed using Lipofectamine^TM^ 2000 (Invitrogen) as per the manufacturer's instructions, lasting for 48 hours.

### 2.5 mRNA and lncRNA real‐time qPCR (RT‐qPCR)

Total RNA from tissue and cells was extracted using the method of TRIzol (No. 15596026, Invitrogen) and reverse‐transcribed into cDNA using PrimeScript^TM^ II 1st Strand cDNA Synthesis Kits (Takara, Dalian, China) as per the manufacturer's protocol. Real‐time PCR was performed with the SYBR green Premix Ex Taq II (Takara) on Applied Biosystems 7500 Real‐Time PCR System (Applied Biosystems, Carlsbad, CA, USA). Data were normalized to the fold change of GAPDH, and relative expression of LINC00160 and TFF3 was determined using the delta‐delta comparative threshold cycle (ΔΔCt) method. Primer information is provided in Table 1.

**Table 1:** Primer sequences for RT‐qPCR

Gene	Sequence (5′‐3′)
LINC00160	F: 5′‐ GATCTACAGCCAACCACCCA‐3′
	R: 5′‐TGCCAAGAATGGCTGAGGTT‐3′
TFF3	F: 5′‐CTCCAGCTCTGCTGAGGAGT‐3′
	R: 5′‐CAGGGATCCTGGAGTCAAAG‐3′
Vimentin	F: 5′‐GACAATGCGTCTCTGGCACGTCTT‐3’
	R: 5′‐TCCTCCGCCTCCTGCAGGTTCTT‐3’
E‐cadherin	F: 5’‐CCCACCACGTACAAGGGTC‐3′
	R: 5′‐CTGGGGTATTGGG GGCATC‐3′
	F: 5′‐CTCGCTTCGGCAGCACA‐3′
U6	R: 5′‐AACGCTTCACGAATTTGCGT‐3′
GAPDH	F: 5′‐ATGGGGAAGGTGAAGGTCGG‐3′
	R: 5′‐GACGGTGCCATGGAATTTGC‐3′

Abbreviations: RT‐qPCR, reverse transcription quantitative polymerase chain reaction; TFF3, trefoil factor 3; GAPDH, glyceraldehyde‐3‐phosphate dehydrogenase‐S.

### 2.6 Western blot analysis

Western blot analysis was performed as early reported^18^ Proteins were incubated with antibodies against TFF3 (1:1000; ab108599; Abcam, Cambridge, MA, USA), C/EBPβ (sc‐150; Santa Cruz Biotechnology, Santa Cruz, CA, USA), Vimentin (1:1000, ab92547), E‐cadherin (1:50, ab1416) and GAPDH (1:2500; ab9485, Abcam), followed by incubation with the secondary antibody, horseradish peroxidase‐labelled goat anti‐rabbit IgG (1:2,000; A0208; Beyotime Institute of Biotechnology, Shanghai, China). Each sample was repeated in triplicate. ImageJ v1.48u software (National Institutes of Health, Bethesda, Maryland, USA) was utilized for quantification analyses.

### 2.7 Subcellular localization

Fluorescence in situ hybridization (FISH) was performed to further examine the subcellular localization of LINC00160.^19^

### 2.8 Fractionation of nuclear/cytoplasmic RNA

The nuclear and cytoplasmic RNA fractions were isolated using a PARIS^™^ kit (Life Technologies, Inc, Gaithersburg, MD, USA) according to the manufacturer's instructions. LINC00160 expression was determined by RT‐qPCR, with U6 as the internal control for nuclear RNA expression and GAPDH for cytoplasmic RNA expression. The primers are shown in Table 1.

### 2.9 Luciferase assay

The putative binding sites of C/EBPβ and TFF3 were obtained by the JASPAR (http://jaspar.genereg.net/). The pmirGLO‐based luciferase reporter plasmids (Promega, Madison, WI, USA) containing wild‐type TFF3, wild‐type TFF3 truncated the putative C/EBPβ binding sites or TFF3 mutated at the putative C/EBPβ binding sites were designed. HEK293T cells (Shanghai Beinuo Biotech Ltd., Shanghai, China) were seeded into 6‐well plates in triplicate and cotransfected with well‐designed pmirGLO‐based reporter plasmids and plasmids carrying C/EBPβ gene using the dual‐luciferase reporter assay system (D0010, Beijing Solarbio Science & Technology Co., Ltd, China). Relative luciferase activity was normalized to that of Firefly luciferase.

### 2.10 Biotinylated RNA pull‐down assays

RNA‐protein interactions were examined using the Pierce™ Magnetic RNA‐Protein Pull‐Down kit (20164, Thermo Fisher Scientific, Waltham, MA, USA). The protein was extracted from the captured RNA‐protein complexes for Western blot analysis. Cell lysates (10 μL) were served as an Input.

### 2.11 RNA immunoprecipitation (RIP)

Enrichment of LINC00160 in the C/EBPβ promoter region was further evaluated using the EZ‐Magna RIP Kit (Millipore, Milan, Italy) according to the manufacturer's instructions. Immunoprecipitated RNA and total RNA from the whole cell lysates (input controls) were extracted for RT‐qPCR analysis.

### 2.12 Chromatin immunoprecipitation (ChIP)

The ChIP assays were performed as previous reported.^20^ The primer with putative C/EBPβ binding sites (positioned at 1360‐1369 bp) was designed as follows: TFF3‐forward sequence was 5′‐TTGAGATTGTGCCACTGCAC‐3′; and TFF3‐reverse sequence was 5′‐CAGCAAGCGGTAAGGGCGGA‐3′. Another pair of primer (served as negative controls) was designed as follows: forward: 5′‐ACAGGCGACAGAACCACCTG‐3′; 5′‐reverse: CCCCAGGCTGCTTCATCCCA‐3′.

### 2.13 Cell survival assays

Cell survival after drug treatment was examined using a CCK‐8 assay as described previously.^21^ Absorbance was read at 490 nm using a microplate reader (Bio‐Rad, Hercules, CA, USA) and used to draw growth curves. The inhibitory rate of cells was calculated based on the formula of [1‐treatment group OD490/control group OD490] × 100%, and the half inhibition concentrations (IC50s) of the cells were also calculated when different drugs were treated.^22^

### 2.14 Clonogenic assay

Clonogenic assays were performed as previously described.^23^

### 2.15 Hoechst 33 342 staining

The chromatin dye Hoechst 33258 was used to observe chromosomal condensation and morphological changes in the nucleus with the fluorescence microscopy (BX50‐FLA; Olympus, Tokyo, Japan). Viable cells showed normal nuclear size with uniform fluorescence. Apoptotic cells displayed condensed nuclei or nuclear condensations.

### 2.16 Migration and invasion assays

Cell migration and invasion assays were carried out using Transwell assays (BD Biosciences, San Jose, CA, USA) according to the manufacturer's instructions.

### 2.17 Flow cytometric analysis

An apoptosis assay was performed using the FITC Annexin V apoptosis detection kit (KeyGen, Nanjing, Jiangsu, China) following the protocol.

### 2.18 Animal studies

A total of 32 BALB/c mice (aged 4‐6 weeks; weighing 18 to 25 g), purchased from Guangdong Medical Laboratory Animal Center, China), were kept under specific pathogen‐free conditions. The licence number for animals used in animal experiments was SYXK (Guangdong) 2018‐0002. MCF‐7/Tax and BT474/Dox cells with stable silencing of LINC00160 or scramble siRNA were resuspended in 50 μL phosphate buffered saline and then added with 50 μL Matrigel till the cell concentration at 5 × 10^6^ cells/mL. Then, the cells were subcutaneously transplanted into each mouse. Mice were injected with paclitaxel (50 mg/kg, iv, 3‐h infusion) or doxorubicin (10 mg/kg, iv, push) once on days 12, 15 and 18 after subcutaneously transplantation. The growth of BC xenografts in mice was monitored every 5 days, and 20 days later, tumour growth was monitored every 3 days. At the 35nd day post‐implantation, the mice were euthanized via carbon dioxide asphyxiation. And immunohistochemistry of xenografts tumours was performed as previously described.^24^

### 2.19 Statistical analysis

Representative experiments were repeated at least three times, and data are expressed as mean ± standard deviation (s.d.). Differences between normally distributed values of two groups were evaluated using Student's *t* test, and those of multiple groups were evaluated using one‐way or two‐way analysis of variance (ANOVA). Pearson correlation coefficients were employed to assess the correlation between the expression of LINC00160 and TFF3. A two‐tailed probability value less than 0.05 was set as the level of significance. All statistics were performed using SPSS 21.0 (IBM Corp. Armonk, NY, USA).

## 3 RESULTS

### 3.1 **Up‐regulated LINC00160 was associated with paclitaxel resistance in BC** ^13^

**Figure 1:**
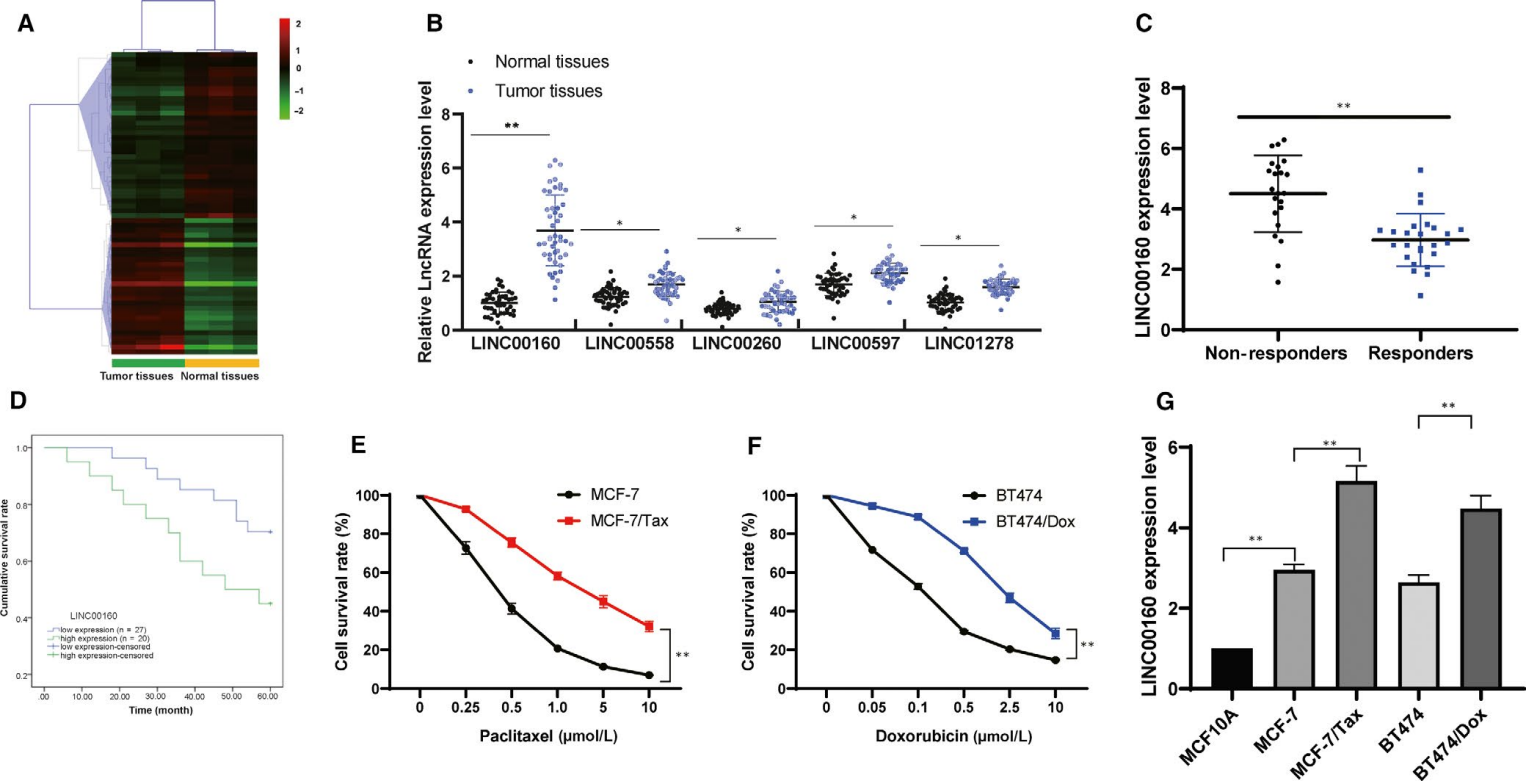
Up‐regulated LINC00160 is associated with chemotherapeutic drug resistance in BC. A, Microarray analysis was performed to determine differentially expressed lncRNAs between BC and adjacent normal tissue. B, RT‐qPCR were performed to determine LINC00160, LINC00558, LINC00260, LINC00597 and LINC01278 expression in 47 BC patients C, LINC00160 expression in paclitaxel‐resistant tumour tissue (n = 22) and in paclitaxel‐sensitive tumour tissue (n = 25) determined using RT‐qPCR. Unpaired *t* test was used for data analysis. D, Kaplan‐Meier survival curves were used to reflect the OS of BC patients. E ~ F, Viability of the parental MCF‐7, MCF‐7/Tax, parental BT474 and BT474/Dox cells detected using CCK‐8 assays. Two‐way ANOVA was used to determine statistical significance. G, LINC00160 expression in MCF10A cells, parental drug‐sensitive MCF‐7 and BT474 cells, and in MCF‐7‐Tax and BT474/Dox cells determined via RT‐qPCR. One‐way ANOVA was used to determine statistical significance. Data are expressed as mean ± s.d., representative of three independent experiments. **P < .01

Three pairs of BC and adjacent normal tissue were used to determine the differentially expressed lncRNAs via microarray analysis. A total of 44 lncRNAs were found to have differential expression between BC and adjacent normal tissue (Figure 1A). The top 5 lncRNAs with most differential expression (LINC00160, LINC00558, LINC00260, LINC00597 and LINC01278) were further validated via RT‐qPCR, which identified that LINC00160 held the greatest differential expression among all lncRNAs in 47 BC patients (Figure 1B). The patients were allocated into responders (to paclitaxel) or non‐responders according to their sensitivity to paclitaxel treatment. RT‐qPCR identified that LINC00160 expression was much higher in the tissue from non‐responders (to paclitaxel) than in those from responders (Figure 1C). Kaplan‐Meier survival curves revealed a reduced OS in BC patients with high expression of LINC00160 compared with those with low expression (Figure 1D). The IC50 values of paclitaxel obtained from dose‐response curves after 72 hours of exposure in the parental MCF‐7 cells and MCF‐7/Tax cells were 0.39 ± 0.04 μmol/L and 4.61 ± 0.25 μmol/L, respectively, and the IC50 values of doxorubicin in the parental BT474 cells and BT474/Dox cells were 0.11 ± 0.02 μmol/L and 2.34 ± 0.16 μmol/L (*P* < .01) (Figure 1E and F). Then, we found that the expression of LINC00160 was increased in BC cells in contrast to MCF10A cells. As we expected, LINC00160 expression was up‐regulated in MCF‐7/Tax and BT474/Dox cells relative to parental MCF‐7 and BT474 cells (*P* < .01) (Figure 1G).

### 3.2 LINC00160 knockdown reduced paclitaxel resistance of MCF‐7/Tax cells and doxorubicin resistance of BT474/Dox cells

**Figure 2:**
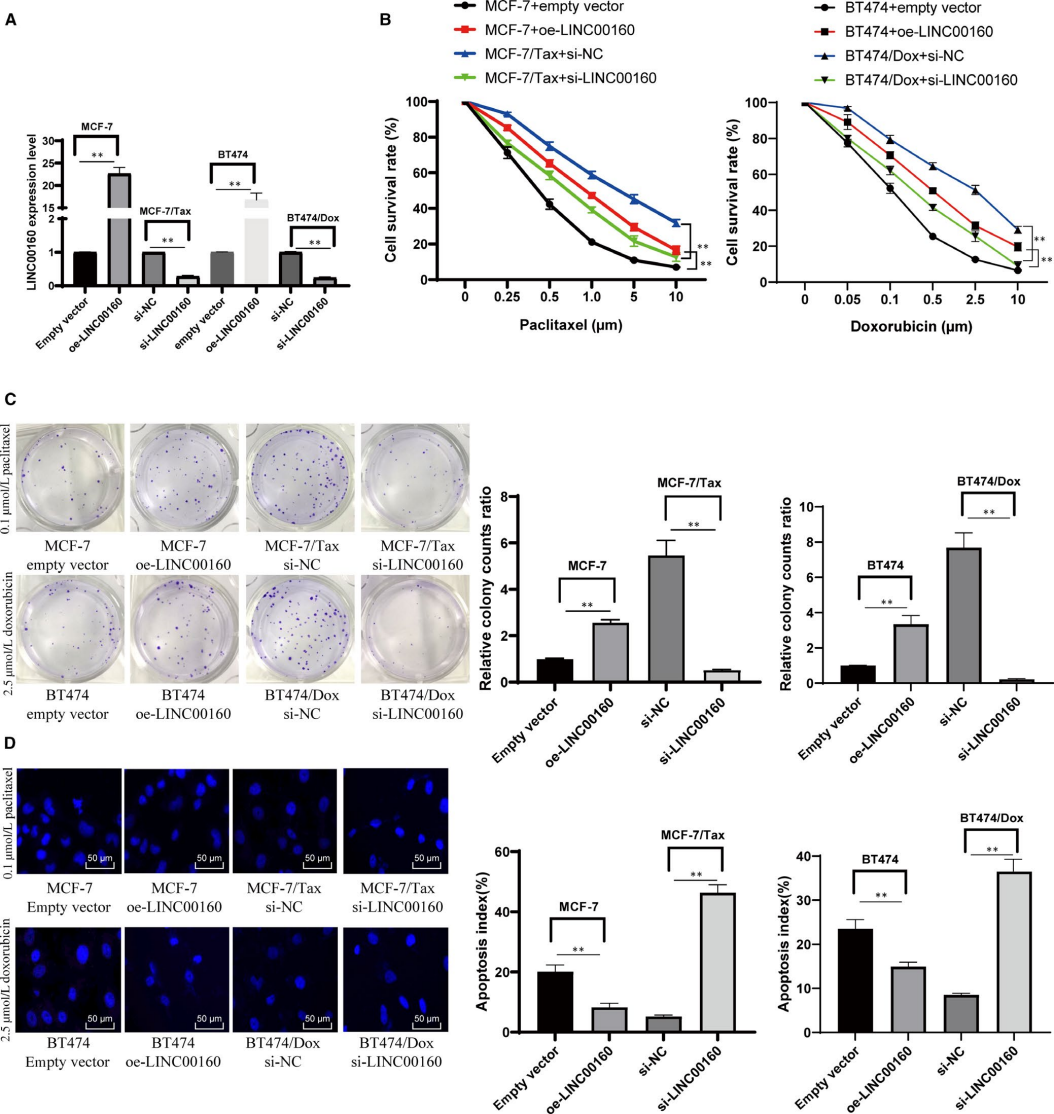
LINC00160 knockdown reduces paclitaxel and doxorubicin resistance in BC cells. A, LINC00160 expression in cells measured by RT‐qPCR. B, Viability and survival rates of parental MCF‐7, MCF‐7/Tax, parental BT474 and BT474/Dox cells measured with CCK‐8 assay. C, Representative views of colonies from parental MCF‐7, MCF‐7/Tax, parental BT474 and BT474/Dox cells via clonogenic assays. D, Representative views of apoptotic cells measured via Hoechst 33258 staining. Data are expressed as mean ± s.d., representative of three independent experiments. Unpaired *t* test and two‐way ANOVA was used. *P < .05 *vs* empty vector group. # < 0.05 *vs* si‐NC group

Next, we constructed LINC00160‐overexpressed parental MCF‐7 and BT474 cells and LINC00160‐depleted MCF‐7/Tax and BT474/Dox cells (Figure 2A). The CCK‐8 results showed that overexpressed LINC00160 increased survival rates of parental MCF‐7 and BT474 cells, and knockdown of LINC00160 resulted in decreased survival rates of MCF‐7/Tax and BT474/Dox cells (Figure 2B). The clonogenic assays (Figure 2C) and Hoechst 33258 staining (Figure 2D) results displayed that LINC00160 overexpression yielded more colonies of parental MCF‐7 and BT474 but less apoptotic cells, while LINC00160 knockdown resulted in decreased cell viability in both MCF‐7/Tax and BT474/Dox cells but increased cell apoptosis (all *P* < .05).

### 3.3 LINC00160 knockdown reduced cell migration and invasion of drug‐resistant cells

**Figure 3:**
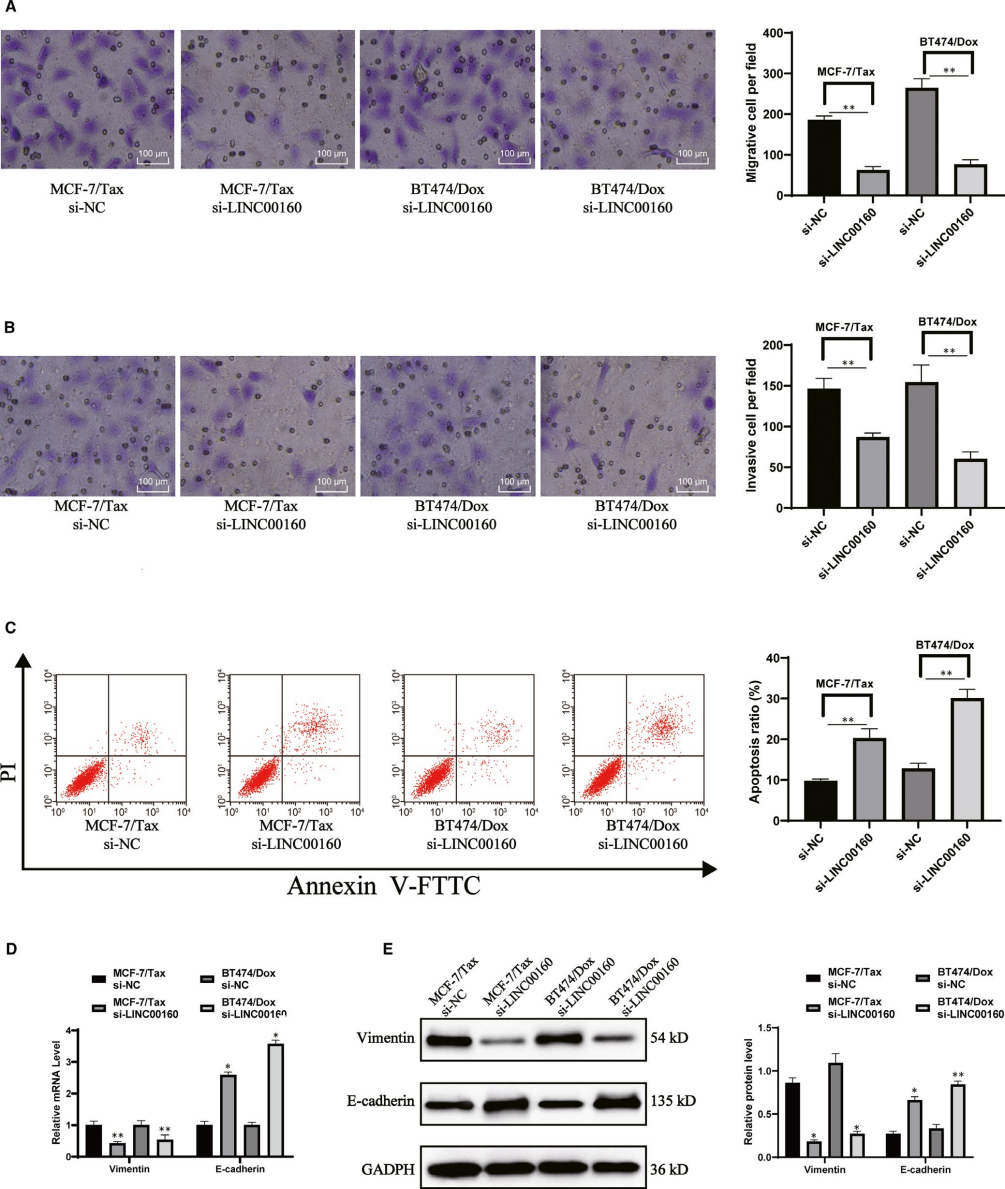
LINC00160 knockdown reduces cell migration and invasion of drug‐resistant cells. A‐B, Migration (A) and invasion (B) of MCF‐7/Tax and BT474/Dox cells measured with Transwell assays. C, MCF‐7/Tax and BT474/Dox cell apoptosis determined by flow cytometry. D‐E, Vimentin mRNA expression (D) and protein levels (E) of Vimentin and E‐cadherin evaluated using RT‐qPCR and Western blot analysis, respectively. Data are expressed as mean ± s.d., representative of three independent experiments. Unpaired *t* test was used to determine statistical significance, *P < .05 vs si‐NC group

The Transwell assay and flow cytometry results found that LINC00160 knockdown reduced cell migration and invasion but induced apoptosis in both LINC00160‐depleted MCF‐7/Tax and BT474/Dox cells (Figure 3A‐C). RT‐qPCR (Figure 3D) and Western blot analysis (Figure 3E) (all *P* < .05) suggested that LINC00160 knockdown led to increased E‐cadherin but decreased Vimentin expression in MCF‐7/Tax and BT474/Dox.

### 3.4 LINC00160 is a nuclear lncRNA

**Figure 4:**
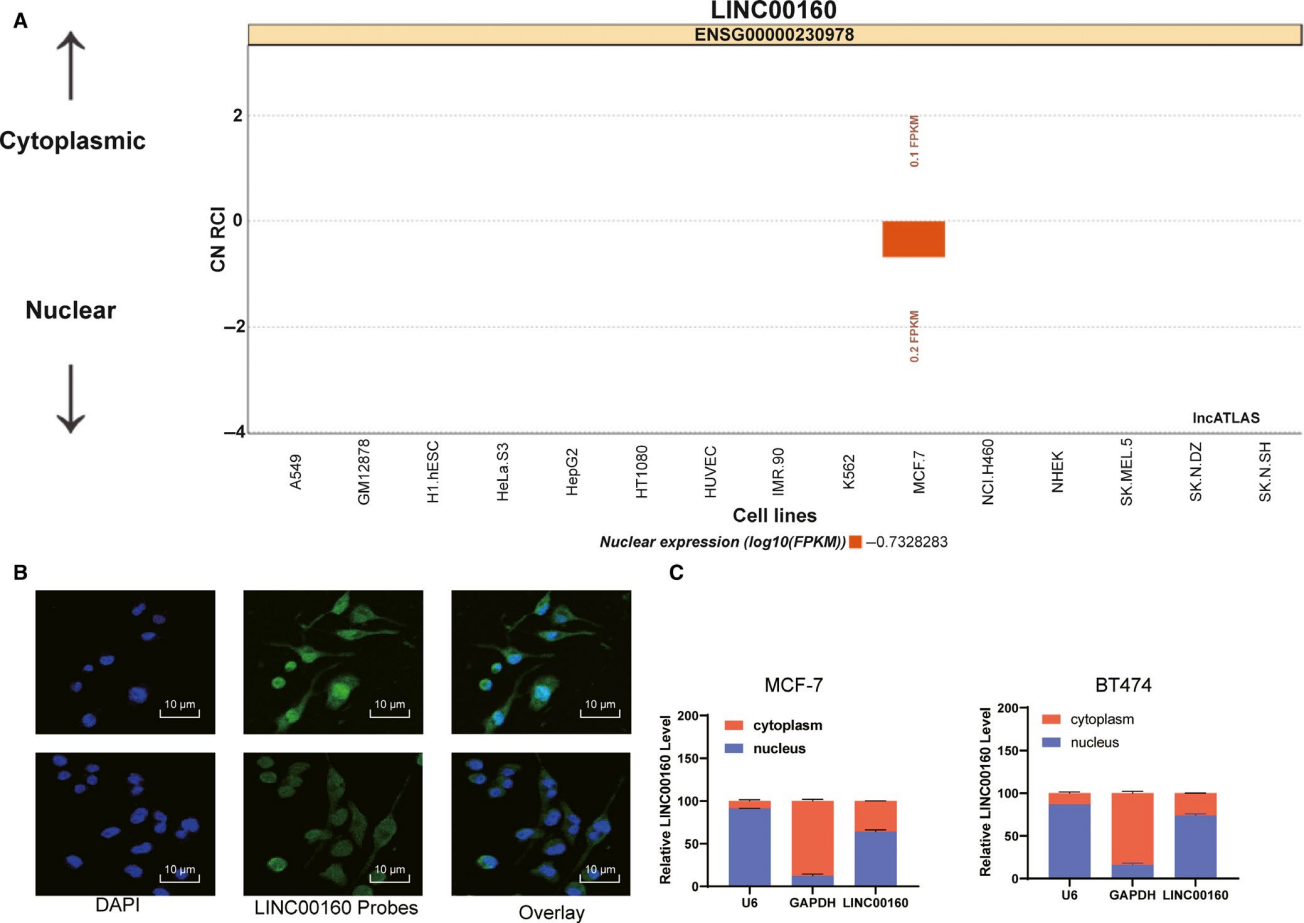
LINC00160 is sublocalized in the nucleus. A, Subcellular localization of LINC00160 in the LncATLAS database. B. FISH experiments with probes targeting LINC00160 were performed to validate the subcellular localization of LINC00160 in MCF‐7 and BT474 cells, LINC00160 were stained with probes targeting LINC00160 (red stain), and the nuclei were stained with DAPI (blue stain). C, Expression of LINC00160 in nuclear and cytoplasmic in MCF‐7 and BT474 cells determined via RT‐qPCR. Data are expressed as mean ± s.d., representative of three independent experiments. One‐way ANOVA was used to determine statistical significance. *P < .05

We firstly searched LncATLAS database for subcellular localization of LINC00160, and the database showed LINC00160 is nucleus‐sublocalized (Figure 4A). FISH experiments with probes targeting LINC00160 were performed to validate the subcellular localization of LINC00160 in MCF‐7 and BT474 cells. As shown in Figure 4B, MCF‐7 and BT474 cells were stained with probes targeting LINC00160 (red stain), and the nuclei were stained with DAPI (blue stain). The merged image showed LINC00160 was nucleus‐sublocalized in MCF‐7 and BT474 cells. In addition, we characterized the nuclear and cytoplasmic expression of LINC00160 by fractionation of nuclear/cytoplasmic RNA and found LINC00160 in the nucleus (Figure 4C).

### 3.5 LINC00160 recruits TF C/EBPβ into the promoter region of TFF3

**Figure 5:**
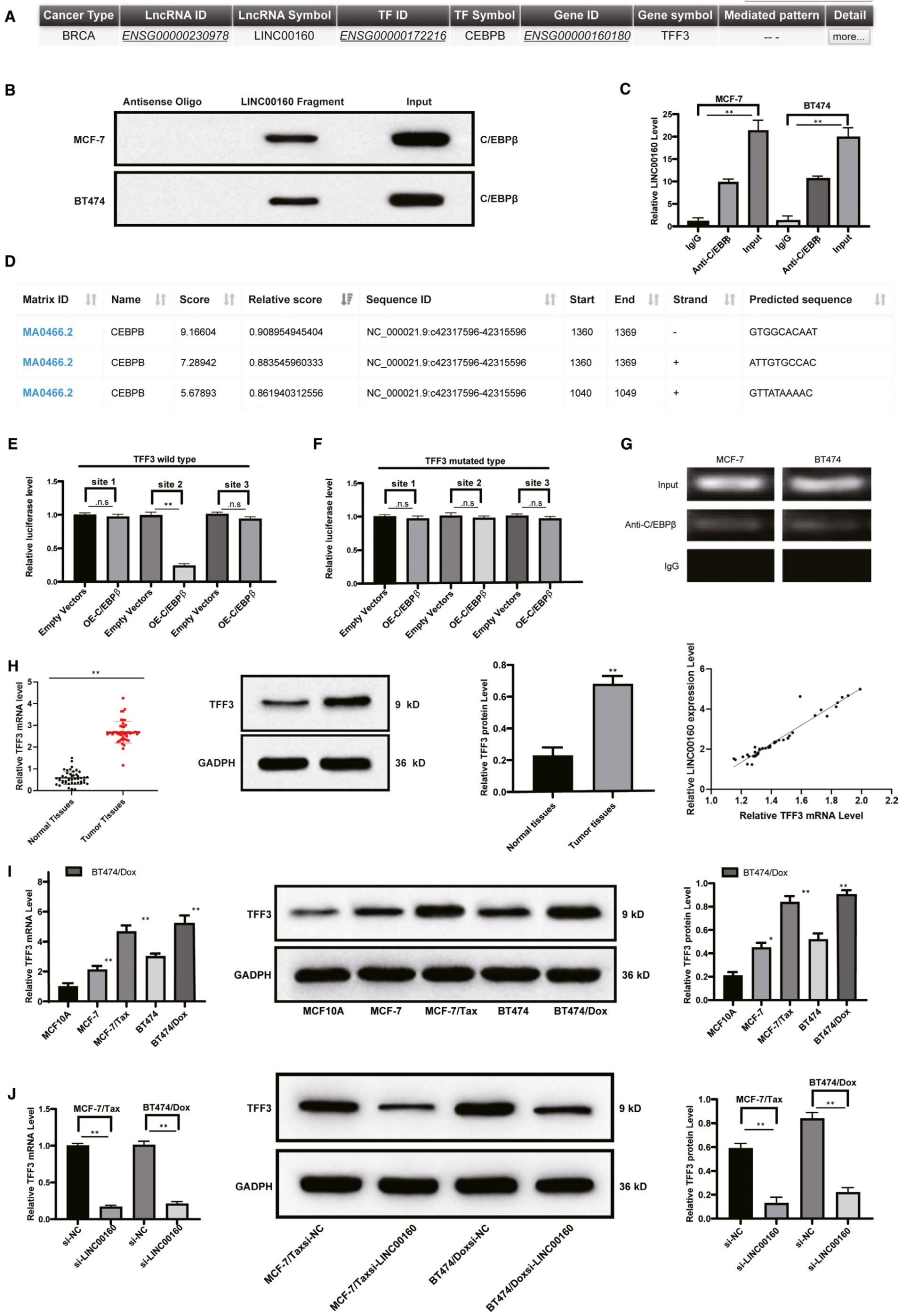
LINC00160 regulates TFF3 *via* C/EBPβ. A, C/EBPβ is a putative TF regulated by LINC00160, and TFF3 is the downstream target gene of C/EBPβ in the LncMAP database. B, C/EBPβ proteins were pulled down by biotinylated RNA fragments of LINC00160 by the Western blot analysis in contrast to antisense oligos. C, Enrichment of LINC00160 on the C/EBPβ promoter region was detected by RIP‐qPCR assay. D, Three binding sites of C/EBPβ and TFF3 obtained from the JASPAR website. E, The Luciferase activity of pmirGLO‐TFF3 with all three C/EBPβ binding sites or pmirGLO‐TFF3, respectively, truncated three C/EBPβ binding sites was determined in the presence of C/EBPβ overexpressing vectors compared with empty vectors. F, The Luciferase activity of pmirGLO‐TFF3, respectively, mutated at three C/EBPβ binding sites was determined in the presence of C/EBPβ overexpressing vectors compared with empty vectors. G, TFF3 was immunoprecipitated in the presence of C/EBPβ antibody relative to IgG by ChIP assays. H‐J, The mRNA expression and protein level of TFF3 in BC tissue and paired adjacent normal tissue (H), in MCF10A cells, parental MCF‐7 cells, MCF‐7/Tax, parental BT474 and BT474/Dox cells (I), and in LINC00160‐depleted MCF‐7/Tax and BT474/Dox cells compared with scramble siRNA‐treated cells were determined by RT‐qPCR and Western blot analyses. J, The mRNA expression and protein level of TFF3. Data are expressed as mean ± s.d., representative of three independent experiments. One‐way ANOVA was used to determine statistical significance. *P < .05

We performed a computer‐based prediction in the LncMAP database (http://bio‐bigdata.hrbmu.edu.cn/LncMAP/), in which C/EBPβ is a putative TF regulated by LINC00160, and TFF3 is the downstream target of C/EBPβ (Figure 5A). We conducted RNA pull‐down assay and RIP assay to confirm the interaction between LINC00160 and C/EBPβ in both MCF‐7 and BT474 cells (Figure 5B‐C). As expected, we found more C/EBPβ proteins were pulled down by biotinylated RNA fragments of LINC00160. More enrichment of LINC00160 on the C/EBPβ promoter region was detected by RIP‐qPCR assay. Next, the focus of experiments shifted to identifying whether TFF3 is a downstream target gene of C/EBPβ. We first performed a computer‐based prediction in the JASPAR website and obtained three binding sites of C/EBPβ and TFF3 (Figure 5D). The results of luciferase activity assays showed that the site 2 was responsible for C/EBPβ binding with TFF3 promoter region (Figure 5E‐F), which was further demonstrated by immunoprecipitation of C/EBPβ and TFF3 in MCF‐7 and BT474 cells. More TFF3 was immunoprecipitated in the presence of C/EBPβ antibody, and increased amplification products of TFF3 were measured in the site 2 (Figure 5G). Subsequently, we found TFF3 was up‐regulated in BC tissue compared with adjacent normal tissue. In 47 BC tissue, the relative expression of TFF3 was found to positively correlate with the expression of LINC00160 (Figure 5H). In addition, TFF3 expression was increased in BC cells compared to MCF10A cells. As expected, TFF3 expression was up‐regulated in MCF‐7/Tax and BT474/Dox cells relative to the parental MCF‐7 and BT474 cells (Figure 5I). In LINC00160‐depleted MCF‐7/Tax and BT474/Dox cells, the expression of TFF3 was found to be decreased (Figure 5J) (all *P* < .05).

### 3.6 TFF3 is responsible for LINC00160‐mediated paclitaxel and doxorubicin resistance in MCF‐7 and BT474 cells

**Figure 6:**
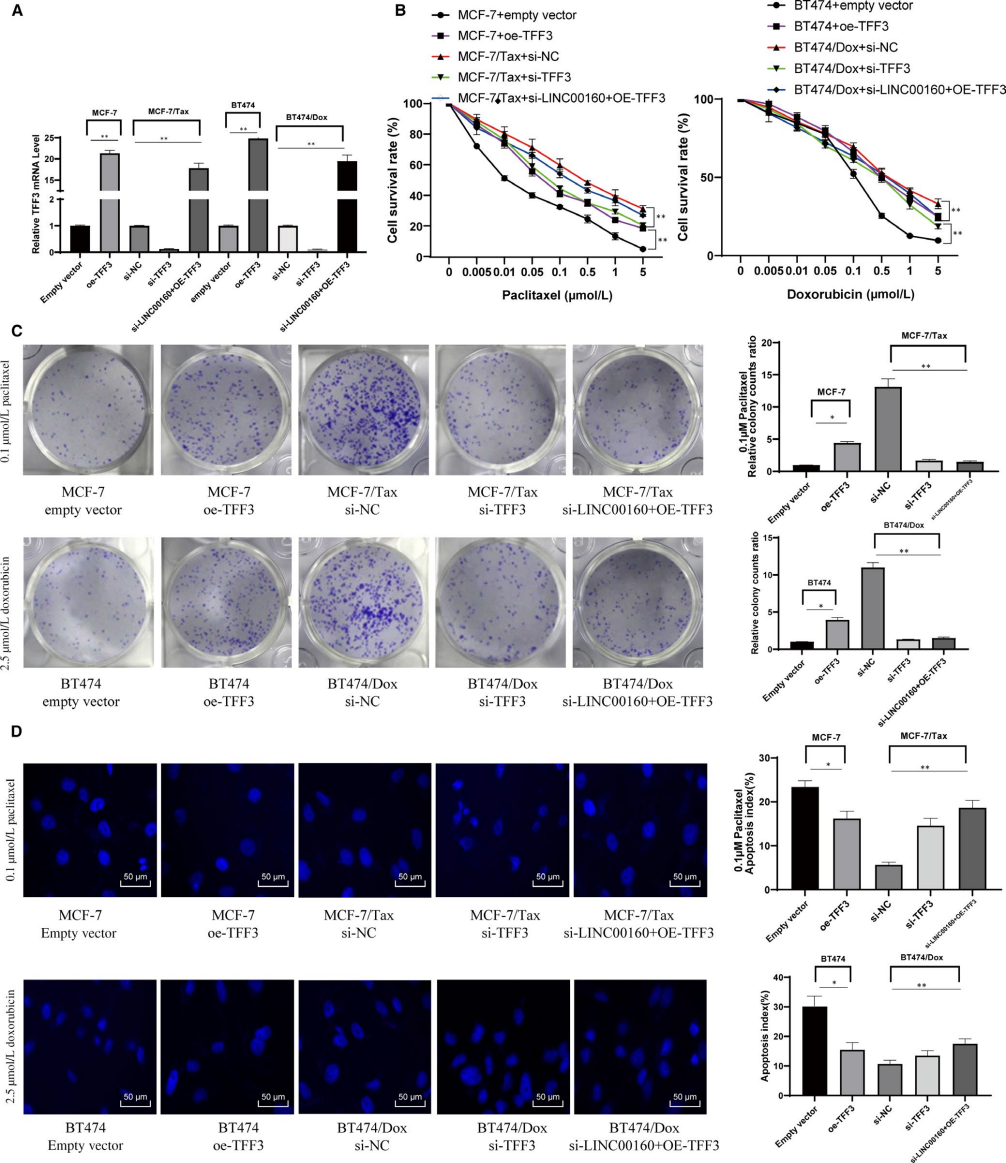
LINC00160 mediates paclitaxel and doxorubicin resistance in MCF‐7 cells by up‐regulating TFF3 *via* C/EBPβ. A, TFF3 expression determined by RT‐qPCR and Western blot analyses, normalized to GAPDH expression. B, Cell viability of parental MCF‐7, MCF‐7/Tax, parental BT474 and BT474/Dox cells measured by CCK‐8 assays. C, Representative views of colonies from parental MCF‐7, MCF‐7/Tax, parental BT474 and BT474/Dox cells evaluated by clonogenic assays. D, Representative views of apoptotic cells from parental MCF‐7, MCF‐7/Tax, parental BT474 and BT474/Dox cells evaluated by Hoechst 33258 staining. One‐way or two‐way ANOVA was used to determine statistical significance. Data are expressed as mean ± s.d., representative of three independent experiments. *P < .05 vs empty vector group; # < 0.05 vs si‐NC group

The effect of TFF3 on paclitaxel resistance in MCF‐7 cells and doxorubicin resistance in BT474 cells was evaluated (Figure 6A). After paclitaxel or doxorubicin treatments, we found overexpressed TFF3 increased survival rates of parental MCF‐7 cells, and knockdown of TFF3 reduced survival rates of MCF‐7/Tax and BT474/Dox cells (Figure 6B). Clonogenic assays showed cell colonies increased in LINC00160‐overexpressed parental MCF‐7 and BT474 cells but reduced in LINC00160‐depleted MCF‐7/Tax and BT474/Dox cells after paclitaxel or doxorubicin treatment (Figure 6C). Hoechst 33258 staining displayed that elevated LINC00160 reduced the apoptosis of parental MCF‐7 cells, whereas LINC00160 knockdown induced the apoptosis of MCF‐7/Tax and BT474/Dox cells (Figure 6D).

### 3.7 LINC00160 knockdown reduces paclitaxel resistance of MCF‐7/Tax cells and doxorubicin resistance of BT474/Dox cells in vivo

**Figure 7:**
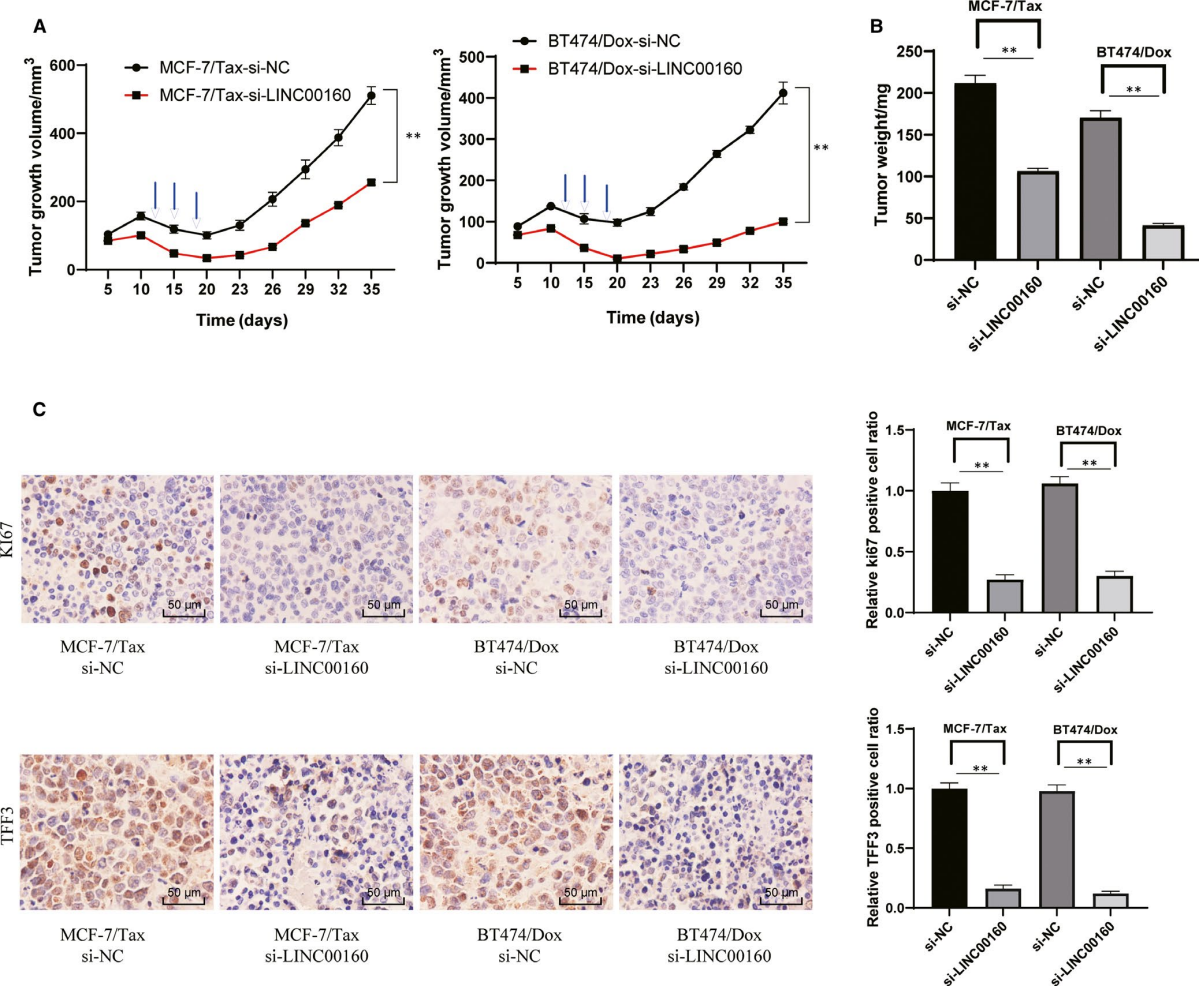
LINC00160 knockdown reduces paclitaxel and doxorubicin resistance of breast cancer cells in vivo. A‐B, Tumour size (A) and tumour weight (B) in nude mice after cell implantation. C, Representative views of TFF3‐ and Ki67‐positive tumour cells and quantification of immunostaining. In panels A and C, two‐way ANOVA was used to determine statistical significance of quantification of immunostaining, whereas in panel B one‐way ANOVA was used. *P < .05 vs si‐NC group

To assess the impact of LINC00160 on paclitaxel and doxorubicin resistance in vivo, we applied a xenograft model in nude mice. Mice transplanted with MCF‐7/Tax or BT474/Dox cells with silencing of LINC00160 showed a decreased tumour growth rate relative to those transfected with scramble siRNA. After being treated with paclitaxel or doxorubicin, tumours in mice transplanted with LINC00160‐depleted cells grew more slowly and were smaller than those in controls (Figure 7A~B). It was shown that LINC00160‐siRNA tumours had less anti‐TFF3 immunostaining and anti‐Ki67 immunostaining than scramble siRNA tumours (Figure 7C).

## 4 DISCUSSION

BC is a frequently diagnosed fatal cancer and the leading cause of cancer‐related death among women.^25^, ^26^ Discovering mechanisms in chemoresistance of anticancer agents has been expected to reduce resistance in clinical practice to favour the long‐term control of metastatic BC.^8^ The lncRNA‐mRNA interaction in tumorigenesis and chemoresistance resistance of cancers, including BC, has aroused wide concerns.^27^, ^28^ Consequently, novel target therapies are required to provide favourable treatment outcomes with less adverse effects. Aberrantly expressed lncRNAs may exert tumour‐suppressive and oncogenic functions by regulating tumour‐related mRNAs upon anticancer therapy.^29^, ^30^ Herein, the current study explored the role of the LINC00160/C/EBPβ/TFF3 axis in BC, and the key findings indicated that LINC00160 mediated the paclitaxel resistance in MCF‐7 cells and doxorubicin resistance in BT474 cells.

LncRNAs have the potential to offer diagnostic, prognostic and predictive functions in BC, with up‐regulated (NEAT1)^12^ or down‐regulated (MAGI2‐AS3)^31^ expression patterns during tumour progression.^32^ The results of this study identified highly expressed LINC00160 in BC tissue and cells. Notably, we found high expression of LINC00160 was related to poor OS. Therefore, it can be concluded that LINC00160 knockdown may suppress BC progression. Furthermore, we found LINC00160 was involved in the resistance of MCF‐7 cells to paclitaxel and BT474 cells to doxorubicin. Specifically, LINC00160 knockdown reduced paclitaxel resistance of MCF‐7/Tax cells and doxorubicin resistance in BT474/Dox cells, as reflected by attenuated cell viability, migration, invasion and stimulated apoptosis. It has been suggested that the resistance to paclitaxel is linked with significant alterations in cell death response accompanied by reduction of various apoptotic factors, which is corresponding to enhancement of the autophagic pathway.^8^ Emerging studies have documented the critical roles of lncRNAs in the chemoresistance of cancers.^33^, ^34^, ^35^ For instance, lncRNA H19 knockdown has been shown to enhance sensitivity of BC cells to paclitaxel *via* the Akt pathway.^36^ Up‐regulated lncRNA BORG has been suggested to augment the survival of BC cells via inducing their resistance to the doxorubicin treatment.^37^ We hypothesized that overexpressed LINC00160 caused significant increases in chemoresistance of BC cells to paclitaxel and doxorubicin both in vitro and in vivo.

Interestingly, we found TFF3 was responsible for LINC00160‐mediated paclitaxel and doxorubicin resistance in MCF‐7 and BT474 cells. LINC00160 regulated TFF3 via C/EBPβ, whereby LINC00160 recruited the TF C/EBPβ into the promoter region of TFF3. A prior lncRNA profile study demonstrated that some mRNAs and lncRNAs are related to the docetaxel resistance in BC cells.^9^ TFF3 has been proposed as an independent risk factor contributing to lymphovascular invasion and lymph node metastasis in BC.^38^ TFF3 renders high sensitivity and specificity to differentiate metastatic BC from the non‐metastatic one.^15^ In addition, TFF3 has also been identified as a sensitive indicator predicting the response of BC cells to endocrine therapy.^39^ It has been suggested that the transcriptional regulation of TFF3 could be realized through promoter binding sites of C/EBPβ.^40^ Partially consistent with our results, Albergaria *et al* found that C/EBPβ isoforms were directly involved in the transcriptional activation of CDH3 in BC.^41^ C/EBPβ can yield distinct biological and regulatory functions, ultimately leading to gene transactivation, playing an important role in mammary gland development and oncogene‐induced breast tumorigenesis.^42^, ^43^ Transcriptional activation of oncogenes may explain the contributory role of C/EBPβ in the development and progression of BC. Resultantly, LINC00160 contributed to the paclitaxel and doxorubicin resistance in MCF‐7 and BT474 cells, respectively, by up‐regulating TFF3 via C/EBPβ in vitro and in vivo.

Taken together, this study provided evidence that overexpressed LINC00160 up‐regulated TFF3 expression through C/EBPβ, whereby potentiating the resistance of MCF‐7 cells to paclitaxel and BT474 cells to doxorubicin. In microarray analysis, 44 lncRNAs were found to have differential expression between BC and adjacent normal tissue (Figure 1A). In the future, we will study the other top 4 lncRNAs with most differential expression (LINC00558, LINC00260, LINC00597 and LINC01278) and estimate whether they are involved in BC cell chemoresistance. Identifying the molecular mechanisms contributing to chemoresistance in BC cells would provide more effective treatment regimens. However, the research is still at the pre‐clinical stage, and the mechanism of action is not yet totally elucidated. Thus, more studies in this area are required in the future to develop clinical values.

## CONFLICTS OF INTEREST

The authors confirm that there are no conflicts of interests.

## AUTHOR CONTRIBUTION

**Huaiguo Wu:** Data curation (equal). **Juan Gu:** Data curation (equal); Formal analysis (equal). **Daoping Zhou:** Data curation (equal); Methodology (equal); Supervision (equal). **Wei Cheng:** Data curation (equal). **Yueping Wang:** Formal analysis (equal); Writing‐original draft (equal); Writing‐review & editing (equal). **Qingping Wang:** Conceptualization (equal); Software (equal). **Xuedong Wang:** Conceptualization (equal); Funding acquisition (equal); Project administration (equal); Supervision (equal); Writing‐original draft (equal); Writing‐review & editing (equal).

## Supporting information

Table S1

## Data Availability

All data generated or analysed during this study are included in this published article.
