# Supplementary material for: LINC00160 mediated paclitaxel‐And doxorubicin‐resistance in breast cancer cells by regulating TFF3 via transcription factor C/EBPβ
Source: J Cell Mol Med. 2020 Jul 11;24(15):8589–602. doi: 10.1111/jcmm.15487 (PMC7412707; doi:10.1111/jcmm.15487)
Supplement: Supplementary file 1 — Table S1 [file JCMM-24-8589-s001.docx]

| Features | Cases (n = 47) |
| --- | --- |
|  |  |
| Age |  |
| ≥50 | 20 |
| <50 | 27 |
| Menstrual condition |  |
| Premenopause | 16 |
| Postmenopause | 31 |
| Tumor size |  |
| ≥2cm | 27 |
| <2cm | 20 |
| TNM staging |  |
| I~II | 22 |
| III~IV | 25 |
| Lymph node metastasis |  |
| Negative | 33 |
| positive | 14 |

**Supplementary Table 1** Clinical characteristics in patients with breast cancer
